# Glutamate in Male and Female Sexual Behavior: Receptors, Transporters, and Steroid Independence

**DOI:** 10.3389/fnbeh.2020.589882

**Published:** 2020-11-24

**Authors:** Vic Shao-Chih Chiang, Jin Ho Park

**Affiliations:** Developmental and Brain Sciences, Department of Psychology, University of Massachusetts Boston, Boston, MA, United States

**Keywords:** sexual behavior, glutamate, steroid-independent, glutamate receptors, glutamate transporters, castration, sexual sluggish

## Abstract

The survival of animal species predicates on the success of sexual reproduction. Neurotransmitters play an integral role in the expression of these sexual behaviors in the brain. Here, we review the role of glutamate in sexual behavior in rodents and non-rodent species for both males and females. These encompass the release of glutamate and correlations with glutamate receptor expression during sexual behavior. We then present the effects of glutamate on sexual behavior, as well as the effects of antagonists and agonists on different glutamate transporters and receptors. Following that, we discuss the potential role of glutamate on steroid-independent sexual behavior. Finally, we demonstrate the interaction of glutamate with other neurotransmitters to impact sexual behavior. These sexual behavior studies are crucial in the development of novel treatments of sexual dysfunction and in furthering our understanding of the complexity of sexual diversity. In the past decade, we have witnessed the burgeoning of novel techniques to study and manipulate neuron activity, to decode molecular events at the single-cell level, and to analyze behavioral data. They pose exciting avenues to gain further insight into future sexual behavior research. Taken together, this work conveys the essential role of glutamate in sexual behavior.

## Introduction

A myriad of reasons motivates human sexual behavior beyond human reproduction, including, but not restricted to, pleasure, expression of love and/or fun, attraction, and reasons apropos to adventure, curiosity, reciprocation, practice, spirituality, etc. (reviewed in Meston and Buss, 2007). In parallel with this, we have only just begun to understand the labyrinth of sexual diversity in terms of characteristics, gender identities, relationship paradigms, and fetishes (reviewed in Gupta, 2012).

Research on sexual behavior benefited greatly from rodent models, including the discovery of several brain regions important for sexual behavior (Snoeren et al., 2014; Rudzinskas et al., 2019). Within these regions, glutamate acts as a pivotal neurotransmitter for sexual behavior (reviewed in Hull and Dominguez, 2006; Dominguez, 2009; Will et al., 2014). These previous reviews on the role of glutamate in male sexual behavior set the roots for the development of the current review. They covered comprehensively the older studies in the field, how sexual experience may involve glutamate, hormonal regulations of glutamate, non-genomic actions of hormones, and glutamate's interactions with other neurotransmitters. Our review builds on this, providing a critical lens on how glutamate, glutamate receptors, and glutamate transporters participate in both male and female sexual behavior. We also expanded with discussions on sexual behavior from perspectives of glial cells, learning and memory, and mechanisms independent of sex steroid hormones. Supplementary to these, this review covered the caveats learned from previous studies as a guideline for optimizing future experiments including novel technologies that researchers can now deploy. Our review also addresses problems of male bias in neuroscience and the need to diversify experimental models.

## Rodent Male Sexual Behavior

Male sexual behavior can be categorized into either appetitive or consummatory behaviors (reviewed in Hull and Dominguez, 2015; Bialy et al., 2019; Le Moëne and Ågmo, 2019). In rodents, this begins with appetitive sexual behavior where the male examines the face and anogenital region of the female (Angoa-Pérez and Kuhn, 2015), followed by the consummatory sexual behaviors of mounting the female's rear and then intromitting several times until they ejaculate (reviewed in Angoa-Pérez and Kuhn, 2015; Hull and Dominguez, 2015; Bialy et al., 2019).

In most rodent species, male sexual behavior highly depends on testosterone, which can then convert into either estrogen or dihydrotestosterone (reviewed in Hull and Dominguez, 2007, 2015). Castration normally abolishes male sexual behavior in rodents, and administration of testosterone reinstates male sexual behavior (reviewed in Dominguez, 2009; Will et al., 2014; Hull and Dominguez, 2015). This is mediated, at least in part, through sex steroid hormones binding to their respective cognate steroid receptors that subsequently induce downstream gene expression related to sexual behavior (reviewed in Balthazart, 2017; Cornil and de Bournonville, 2018). Several brain regions orchestrate male sexual behavior, including the medial preoptic area (mPOA), the parvocellular sub-parafasicular thalamic nucleus, the paraventricular nucleus (PVN), the caudodorsal part of the posteromedial part of the bed nucleus of the stria terminalis, the posterodorsal part of the medial amygdala (MeA), and the brainstem (reviewed in Merari and Ginton, 1975; Kondo et al., 1990; Sakamoto, 2012; Snoeren et al., 2014).

In addition to sex steroid hormones driving sexual behavior, steroid-independent mechanisms likewise play a significant role in regulating sexual behavior in numerous mammalian species. For example, a significant proportion of male hybrid BD62F1 mice exhibit persistent male sexual behavior long after castration, in which ~30% of the males continued to exhibit male sexual behavior at least 20 weeks post-castration (McGill and Tucker, 1964; Clemens et al., 1988). Other examples include Siberian hamsters and deer mice, of which after castration, gonadectomized males demonstrated male sexual behavior for at least 19 weeks (Park et al., 2004) and 13 weeks (Clemens and Pomerantz, 1981), respectively.

### Glutamate and Rodent Male Sexual Behavior

**Figure 1:**
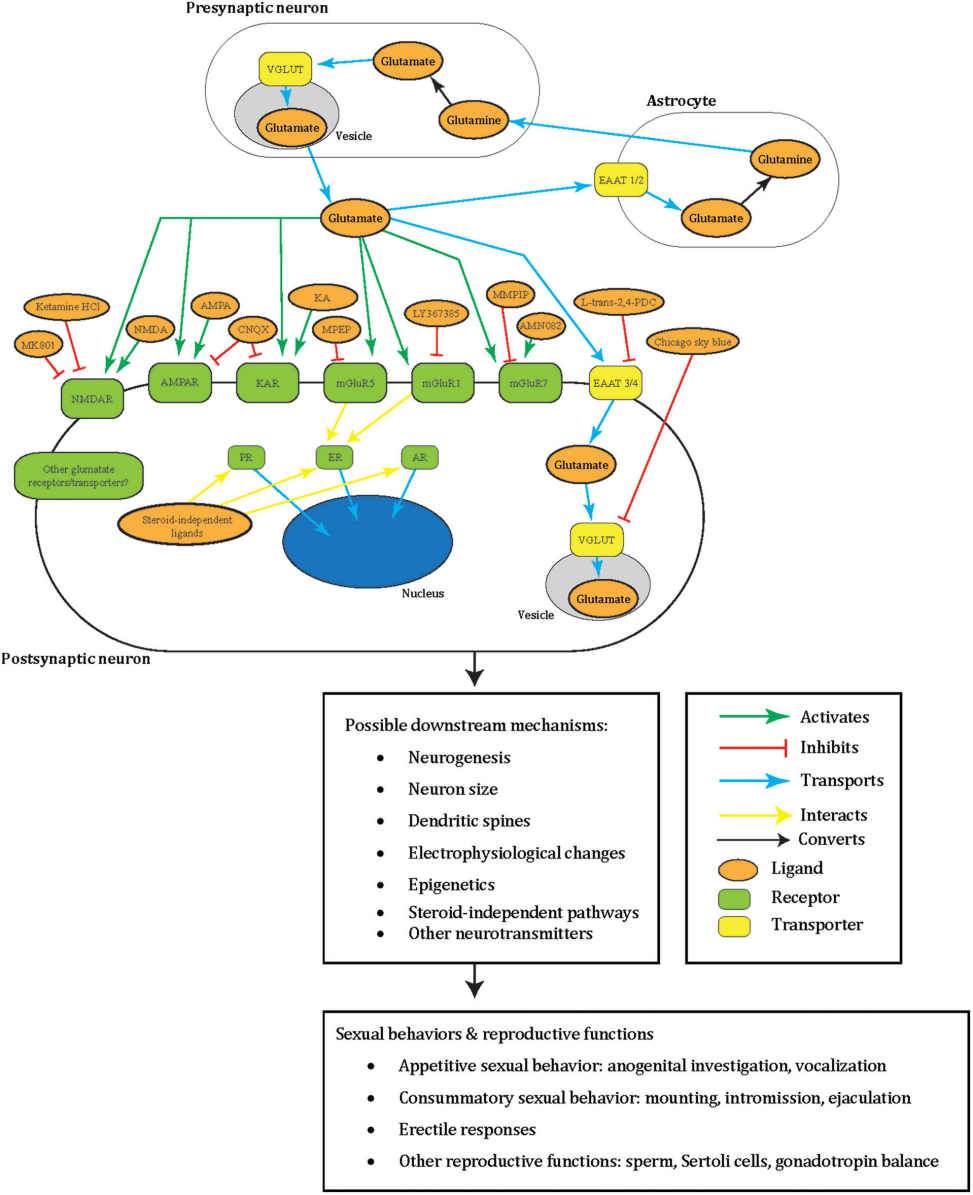
A summary illustrating how glutamate may facilitate male sexual behavior in a tripartite synapse between pre- and post-synaptic neurons and astrocyte. Glutamate (orange oval) is released from the synaptic vesicles (gray oval) of the pre-synaptic neuron (white oblong). These act as agonists (green arrow) to the post-synaptic neuron (white oblong) glutamate receptors (green oblong) in male sexual behavior: NMDAR, AMPAR, KAR, mGluR5, mGluR1, mGluR7, or other receptors yet to be elucidated. Glutamate can also be transported (blue arrow) by glutamate transporters EAAT 3/4 (yellow oblong) across the neuron cell membrane or into synaptic vesicles (gray oval) by VGLUT (yellow oblong) in male sexual behavior, or other transporters not tested. Additionally, to prevent excitotoxicity, extracellular glutamate transports (blue arrow) into astrocytes (white oblong) by astrocytic EAAT 1/2 (yellow oblong) and converts (black arrow) into non-toxic glutamine (orange oval). The glutamine can then release into the extracellular space and taken up by neurons that synthesize glutamate again. Glutamatergic signaling was tested with male sexual behavior using different ligands (orange oval) that are either antagonists (green arrow) or agonists (red blunted arrow) to glutamate receptors or transporters. Areas these cells may locate in the brain include the medial preoptic area, medial preoptic nucleus, NAc, paraventricular nucleus, posterodorsal part of the medial amygdala, and sexually dimorphic nucleus-preoptic area. Intracellular sex steroid receptors (green oblong) are often implicated in male sexual behavior and traditionally viewed to activate by sex steroid hormones. Once these receptors activate, they transport (blue arrow) into the nucleus (blue oval) to act as transcription factors. ER interacts (yellow arrow) with mGluR5 and mGluR1, and interactions of other glutamate receptors with these sex steroid receptors including non-steroid ligands awaits to be explored. All of these intracellular events may lead to possible downstream changes including in neurogenesis, neuron size, dendritic spines, electrophysiology, epigenetics, other neurotransmitters, and steroid-independent pathways. These may subsequently result in male sexual and reproductive behavior including appetitive sexual behavior (anogenital investigation, vocalization), consummatory sexual behavior (mounting, intromission, ejaculation), erectile responses, and other reproductive functions (sperm, Sertoli cells, gonadotropin balance). Please note that the complexity of this glutamatergic signaling is composed of the several redundant possible molecular mechanisms based on what has been tested in males so far. These have yet to be elucidated for their specific molecular effects. The same redundancy also goes to the downstream changes at the cellular, circuit, organ, and behavioral level, which remain a black box of how activation of specific GluRs go from specific whole-cell changes to specific sexual behavior. This dilemma is shared with females, and therefore several of the depicted mechanisms appear repetitive. NMDAR, NMDA receptor; AMPA, AMPA receptor; KAR, kainate receptor; EAAT, excitatory amino acid transporter; PR, progesterone receptor; ER, estrogen receptor; AR, androgen receptor; MMPIP, NAM 6-(4-methoxyphenyl)-5-methyl-3-pyridinyl-4-isoxazolo[4,5-c]pyridin-4(5H)-one.

**Figure 2:**
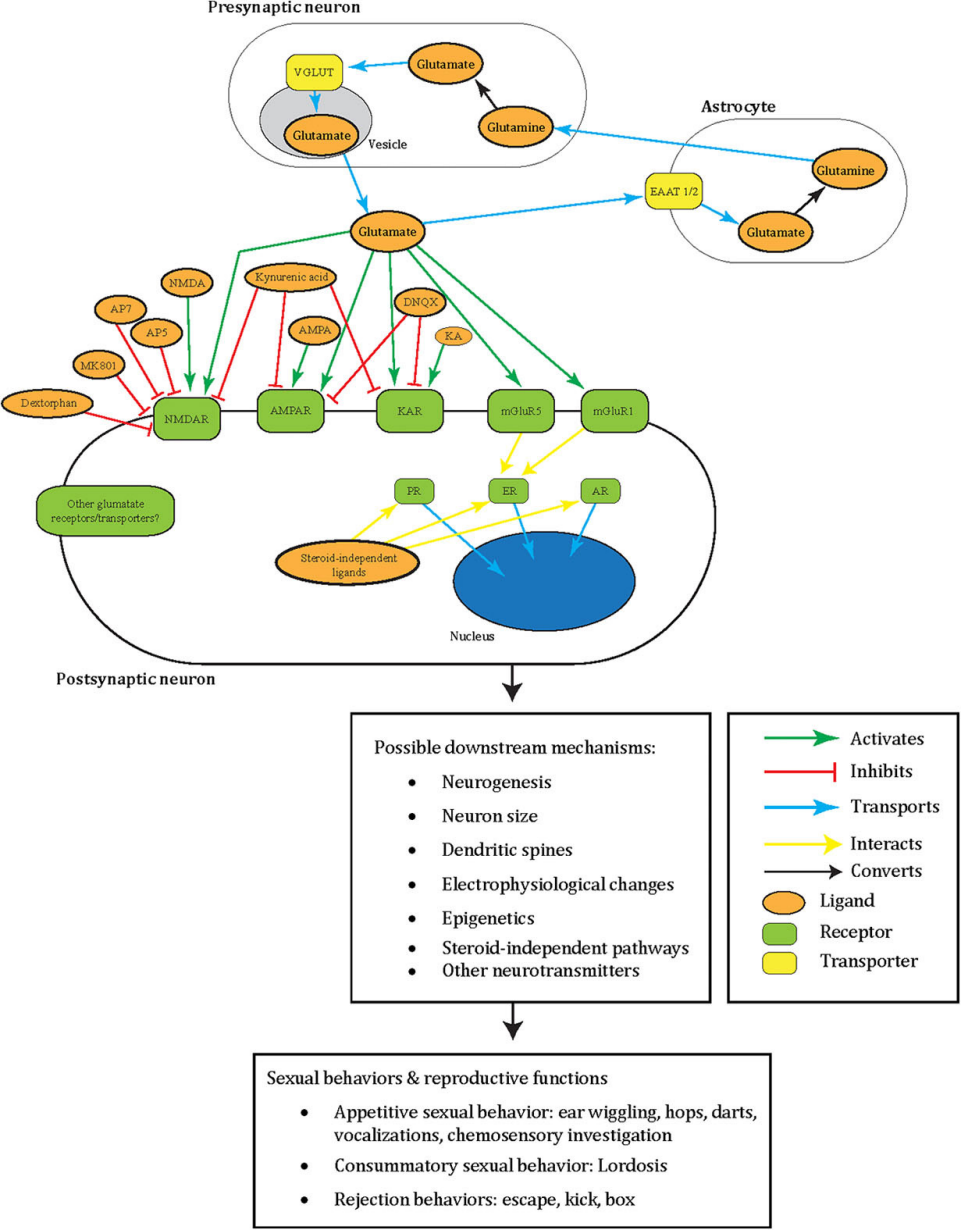
A summary illustrating how glutamate may facilitate female sexual behavior in a tripartite synapse between pre- and post-synaptic neurons and astrocyte. Glutamate (orange oval) is released from the synaptic vesicles (gray oval) of the pre-synaptic neuron (white oblong). These act as agonists (green arrow) to the post-synaptic neuron (white oblong) glutamate receptors (green oblong) in female sexual behavior: NMDAR, AMPAR, KAR, mGluR5, mGluR1, or other receptors yet to be elucidated. Glutamate can also be transported into synaptic vesicles (gray oval) by VGLUT (yellow oblong). Additionally, to prevent excitotoxicity, extracellular glutamate transports (blue arrow) into astrocytes (white oblong) by astrocytic EAAT 1/2 (yellow oblong) and converts (black arrow) into non-toxic glutamine (orange oval). The glutamine can then release into the extracellular space and taken up by neurons that synthesize glutamate again. Glutamatergic signaling was tested with female sexual behavior using different ligands (orange oval) that are either antagonists (green arrow) or agonists (red blunted arrow) to glutamate receptors. Areas these cells may locate in the brain include the ventromedial hypothalamus, mPFC, NAc, VTA, mediobasal hypothalamus, septum, and preoptic area. Intracellular sex steroid receptors (green oblong) are often implicated in female sexual behavior and traditionally viewed to activate by sex steroid hormones. Once these receptors activate, they transport (blue arrow) into the nucleus (blue oval) to act as transcription factors. ER interacts (yellow arrow) with mGluR5 and mGluR1, and interactions of other glutamate receptors with these sex steroid receptors including non-steroid ligands await to be explored. All of these intracellular events may lead to possible downstream changes including in neurogenesis, neuron size, dendritic spines, electrophysiology, epigenetics, other neurotransmitters, and steroid-independent pathways. These may subsequently result in female sexual and reproductive behavior including appetitive sexual behavior (ear wiggling, hops, darts, vocalization, chemosensory investigation), consummatory sexual behavior (lordosis), and rejection behaviors (escape, kick, box). Please note that the complexity of this glutamatergic signaling is composed of the several redundant possible molecular mechanisms based on what has been tested in females so far. These have yet to be elucidated for their specific molecular effects. The same redundancy also goes to the downstream changes at the cellular, circuit, organ, and behavioral level, which remain a black box of how activation of specific GluRs go from specific whole-cell changes to specific sexual behavior. This dilemma is shared with males, and therefore several of the depicted mechanisms appear repetitive. NMDAR, NMDA receptor; AMPA, AMPA receptor; KAR, kainate receptor; PR, progesterone receptor; ER, estrogen receptor; AR, androgen receptor; MMPIP, NAM 6-(4-methoxyphenyl)-5-methyl-3-pyridinyl-4-isoxazolo[4,5-c]pyridin-4(5H)-one.

Glutamate is a critical excitatory neurotransmitter in the vertebrate nervous system (reviewed in Fonnum, 1984) and is associated with male sexual behavior (reviewed in Hull and Dominguez, 2006; Dominguez, 2009; Will et al., 2014). We summarized the role of glutamate in sexual behavior we covered in this review in Figure 1 for males and Figure 2 for females. In the sexual dimorphic nucleus-POA and lateral posterodorsal MeA of male gerbils, 25% of Fos proto-oncogene (FOS)-positive cells (immediate early gene marker of neuron activation) colocalized with glutamate 1.5 h after mating (Simmons and Yahr, 2003). Later, microdialysis experiments measured glutamate release from the mPOA and PVN of male rats during mounting, intromission, and ejaculation (Melis et al., 2004; Dominguez et al., 2006; Hull and Dominguez, 2007).

While we recognize the importance of these microdialysis studies, this method biases toward measuring glutamate release of non-synaptic sources. Future studies may address this by utilizing microsensors, which measure glutamate from synaptic sources (reviewed in van der Zeyden et al., 2008). Another limitation of microdialysis pertains to its inability to uncover the source of the glutamate release. To disentangle this in sexual behavior, researchers can deploy techniques such as [^3^H] D-aspartate and anterograde labeling (Dominguez et al., 2004, 2006). Despite these limitations, the studies provided strong evidence that glutamate release is implicated in male sexual behavior.

Early glutamate studies helped establish a relationship with male sexual behavior, but they did not unravel whether glutamate poses as a cause, consequence, or just a correlated change in this behavior. In order to ascertain the causality of glutamate in male sexual behavior, studies directly administered glutamate in animal models. One study found an increased erectile function with a 0.5-nmol glutamate infusion to the mPOA of male Sprague–Dawley rats, as measured through the intracavernous pressure of the penis (Giuliano et al., 1996). Although we acknowledge this erectile response functions not as male sexual behavior *per se*, it does predict the success of the subsequent sexual repertoire; however, this surpasses the scope of this review. For those interested, we refer to a recent review that looked at glutamate studies with male reproductive functions including sperm abnormality, oxidative damage, altered Sertoli cells, and impaired gonadotropin imbalance (reviewed in Kayode et al., 2020).

### Glutamate Receptors and Rodent Male Sexual Behavior

#### Association With Glutamate Receptors

In conjunction with examining glutamate studies on how they engage in male sexual behavior, unraveling glutamate receptors (GluRs) provide a more comprehensive view of this narrative. A major class of GluRs comprises of ionotropic GluRs, which encompass NMDA, AMPA, and KA receptors (reviewed in Tzingounis and Wadiche, 2007). They function as ion channels for sodium, potassium, and calcium ions to facilitate excitatory postsynaptic responses (reviewed in Tzingounis and Wadiche, 2007). They achieve this through their central pore generally made up of four large subunits. For NMDA receptors, these form heterotetramers made up of two GluN1 subunits and two GluN2 subunits, all of which are composed of a large extracellular domain, transmembrane components, a pore loop and an intracellular domain (reviewed in Giacometti and Barker, 2020). The subunits have structural differences such that GluN1 mainly binds to glutamate and GluN2 modulates activity. In terms of AMPA receptors, they form kindred heterotetrameric structures mostly with dimers of GluA2 added with other subunits GluA1, GluA3, or GluA4 (reviewed in Tzingounis and Wadiche, 2007). These subunits interact with different scaffold proteins due to their variations in the C-terminal sequence. In the case of KA receptors, scientists have discovered five types of receptor subunits GRIK1-5 so far, and they consist of extracellular domains, a binding cleft, a transmembrane region, a p-loop and a cytoplasmic region (reviewed in Giacometti and Barker, 2020).

In a study on male Sprague–Dawley rats, many FOS-positive neurons in the mPOA during mating co-expressed with GluN1 (immunohistochemistry), and these GluN1 exhibited increased phosphorylation (Western blot), which indicates the activation of these receptors (Dominguez et al., 2007). A factor to consider for the future as mentioned by Dominguez et al. (2007) concerns the choice of antibodies. Studies can begin with polyclonal antibodies to probe the participation of its target and then follow with monoclonal antibodies that determine where the phosphorylation occurs. For example, in the case of GluN1, Ser896, and Ser897 present as potential sites of phosphorylation among others (Muniz and Isokawa, 2015). Phosphorylation of different sites can lead to differential effects on sexual behavior. Furthermore, receptor trafficking dynamics of these GluRs during sexual behavior may offer new avenues of research. These receptor dynamics can range from short to long distances between cellular compartments and interact with other proteins and extracellular elements (reviewed in Groc and Choquet, 2020). This may have significant implications on sexual behavior due to the importance of these mechanisms for neurotransmission.

#### Manipulating NMDA Receptors

In addition to identifying a role of GluN1 in male sexual behavior to provide insight into the causal role of glutamate on male sexual behavior, studies that directly manipulate GluRs through pharmacological agents are also valuable in providing further understanding of causality. In terms of NMDA receptor antagonists, several drugs exist, consisting of uncompetitive antagonists (e.g., AP5 and AP7) that bind to the same site as glutamate, uncompetitive channel blockers (e.g., ketamine and MK-801) that block the channel directly, and kynurenic acid that acts on the glycine binding site of the receptor (reviewed in Tzingounis and Wadiche, 2007).

Administration of MK801, either systemically or via microinjections into the brain (mPOA and PVN), diminished male rat sexual behavior (Fleming and Kucera, 1991; Powell et al., 2003; Melis et al., 2004; Dominguez et al., 2007; Vigdorchik et al., 2012; Beloate et al., 2016). In Table 1, we summarized the above studies, as well as subsequent studies reviewed. Male rodents also make ultrasonic vocalization as an appetitive sexual behavior, and 50 kHz calls were reported during male sexual behavior tests (Bialy et al., 2000). MK801 administration (0.6 μg) to the preoptic-anterior hypothalamus in male Wistar rats reduced 50-kHz calls (Brudzynski and Pniak, 2002). In contrast to these studies, infusions of 1 or 2 μg of MK801 to the NAc for 4 consecutive days did not change sexual behavior in male Sprague–Dawley rats (Beloate et al., 2016). The different brain regions targeted may explain this discrepancy between these studies. In addition to MK801, one study administered ketamine HCl systemically to sexually exhausted rats (those with no interest in stimulus females after the previous mating) and found all concentrations of ketamine below 1 mg/kg increased the percentage of males that resumed ejaculation (Rodríguez-Manzo, 2015).

**Table 1:** Effects of glutamate, glutamate receptor antagonists and agonists, and glutamate transporter inhibitors on male sexual behavior.

**Male species of strain**	**Drug and dose**	**Administration**	**Sexual behavior assessed**	**Sexual behavior changed**	**Comparison sample**	**References**
Rat (SD), adult, mated for 4 consecutive days	1 or 2 μg MK801 (NMDA receptor antagonist)	NAc, bilateral, 4 consecutive days, 15 min before test each day	LT to MT, IO, EJ. 30 min test	No changes	Saline	Beloate et al., 2016
Rats (W), adult, did not specify sexual experience	0.6 μg MK801 (NMDA receptor antagonist)	Preoptic-anterior hypothalamic, bilateral, 5 min before test	Vocalizations at 10–180 kHz. 10 min test	↓ 50 kHz calls	Saline	Brudzynski and Pniak, 2002
Dorper rams, 2–3 years old, did not specify sexual experience	7 mg/kg live weight of glutamate	Im, every 4 days for 30 days, did not specify injection time	# of appetitive sexual behavior and consummatory sexual behavior	↑ # of appetitive sexual behavior and consummatory sexual behavior	Saline	Calderón-Leyva et al., 2018
Rats (LE), sexually experienced	Mixture of 250 μM Chicago sky blue, 250 uM L-trans-2,4-pPDC (glutamate transporter inhibitors)	mPOA, unilateral, did not specify reverse dialysis time before testing	LT to 1st MT, 1st IO, 1st EJ. PEI. # of total MT, IO, EJ. Did not specify test time	↑ # of EJ; ↓ LT to EJ; ↑ PEI	ACSF	Dominguez et al., 2006
Rats (SD), adult, sexually experienced	1.25 μg MK801 (NMDA receptor antagonist)	mPOA, bilateral, 15 min before test	# of MT, IO, EJ; LT to MT, IO, EJ; PEI. 1-h test	↓ # of EJ; ↑ LT to EJ, PEI, # of MT	Saline	Dominguez et al., 2007
Rats (W), sexually sluggish	1, 2.5, 5 mg/kg KA	Ip, 1 h before testing	LT of MT, IO, EJ; # of MT, IO, EJ. 30 min test	1 and 2.5 mg/kg: ↓ LT to MT and IO; ↑ # of MT and IO. 5 mg/kg: ↑ LT to MT and EJ; ↓ # of MT and IO	Saline	Drago and Busǎ, 1997
Rats (W), good copulators	1, 2.5, 5 mg/kg KA	Ip, 1 h before testing	LT of MT, IO, EJ; # of MT, IO, EJ. 30 min test	5 mg/kg: ↑ LT to MT, IO, EJ; ↑# to MT, IO, EJ	Saline	Drago and Busǎ, 1997
Rats (W), sexually sluggish	1, 2.5, 5 mg/kg KA	Ip, 20 days before testing, injection time 1 h before 1st test.	LT of MT, IO, EJ; # of MT, IO, EJ. 30 min test	5 mg/kg: ↑ LT to MT, IO, EJ; ↓ # to MT, IO, EJ	Saline	Drago and Busǎ, 1997
Rats (W), good copulators	1, 2.5, 5 mg/kg KA	Ip, 20 days before testing, injection time 1 h before 1st test	LT of MT, IO, EJ; # of MT, IO, EJ. 30 min test	5 mg/kg: ↑ LT to MT, IO, EJ	Saline	Drago and Busǎ, 1997
Rats (SD), adult, did not specify sexual experience	0.5 nmol L-glutamate	Posterior mPOA, unilateral, 5 min before test	Erectile response: LT and filling rate of the corpus cavernosum; intracavernous pressure/blood pressure ratio. 8 min test	↑ intracavernous pressure	Same rat before injection	Giuliano et al., 1996
Rats (LE), sexually experienced.	1 and 3 mg/kg LY379268 (mGluR2/3 agonist)	Sex seeking; LT to MT, IO, EJ; # of MT, IO. PEI. 30 min test	LT to MT, IO, EJ; # of MT, IO. PEI. 30 min test	No changes	Vehicle	Li et al., 2013
Rats (LE), sexually experienced.	20 and 10 mg/kg MPEP (mGluR5 antagonist)	Sex seeking; LT to MT, IO, EJ; # of MT, IO. PEI. 30 min test	LT to MT, IO, EJ; # of MT, IO. PEI. 30 min test	All concentrations: ↓ sex-seeking. 20 mg/kg: ↑ LT to MT, IO, EJ; ↑ PEI; ↓ # of MT and IO	Vehicle	Li et al., 2013
Rats (LE), sexually experienced.	3, 10, 20 mg/kg AMN082 (mGluR7 antagonist)	Sex seeking; LT to MT, IO, EJ; # of MT, IO. PEI. 30 min test	LT to MT, IO, EJ; # of MT, IO. PEI. 30 min test	10 and 20 mg/kg: ↓ sex-seeking. 10 mg/kg: ↑ LT to EJ; ↑ PEI; ↓ # of MT; 20 mg/kg: ↑ LT to MT, IO, EJ; ↑ PEI; ↓ # of MT and IO	Vehicle	Li et al., 2013
Mice (C57), sexually naïve, 12–24 weeks old	1.25 μg MMPIP (mGluR7 antagonist)	BNST, did not specify laterality, 60 min before test	MT intruder male (similar to trying to induce lordosis of females). 15 min test	↑% of mice MT	DMSO	Masugi-Tokita et al., 2016
Mice (C57), sexually naïve, 12–24 weeks old	1.25 μg MMPIP (mGluR7 antagonist)	MeA, did not specify laterality, 60 min before test	MT intruder male (similar to trying to induce lordosis of females). 15 min test	No changes	DMSO	Masugi-Tokita et al., 2016
Mice (C57), sexually naïve, 12–24 weeks old	1.25 and 3.75 μg MMPIP (mGluR7 antagonist)	Lateral ventricle, did not specify laterality, 60 min before test	MT intruder male (similar to trying to induce lordosis of females). 15 min test	No changes	DMSO	Masugi-Tokita et al., 2016
Rats (LE), sexually naïve	0.1, mg/kg MK801 (NMDA receptor antagonist)	Ip, 15 min before testing	LT to MT, IO, EJ; PEI; # of MT, IO, EJ. LT and # to MT in 1st EJ. Total IO ratio. 30 min test	↓ # of EJ; ↓ of IO ratio; ↑ LT to IO and EJ	Saline	Powell et al., 2003
Rats (LE), 1 sexual experience	0.05, 0.1, 0.2 mg/kg MK801 (NMDA receptor antagonist)	Ip, 15 min before testing	LT to MT, IO, EJ; PEI; # of MT, IO, EJ. LT and # to MT in 1st EJ. Total IO ratio. 30 min test	0.2 mg/kg: ↓ # of EJ; ↓ IO ratio; ↑ # of MT. 0.1 mg/kg: ↑ # of MT	Saline	Powell et al., 2003
Rats (LE), sexually naïve exposed to female for 30 min, but not allowed to copulate (7 days)	0.2 mg/kg MK801 (NMDA receptor antagonist)	Ip, 20 min before exposure to female	LT to MT, IO, EJ; PEI; # of MT, IO, EJ. LT and # to MT in 1st EJ. Total IO ratio. 30 min test	↑ LT to IO and EJ; ↓ # of EJ	Saline	Powell et al., 2003
Rats (W), adult, sexually experienced, and sexually exhausted.	0.01–3.0 mg/kg ketamine HCl (NMDA receptor antagonist)	Ip, 30 min before test	% of males MT, IO, EJ, resumed copulation. LT to IO, EJ; # of MT, IO; PEI. 30 min test	All concentration below 1.0 mg/kg: ↑ % of males MT, IO, EJ, resumed copulation. 1.0 mg/kg: ↑ % of males MT, IO, EJ. 3.0 mg/kg: ↑ % of males MT and IO. 0.01 mg/kg: ↓ # of IO. 0.1 mg/kg: ↑ PEI. 0.3 mg/kg: ↑ # of MT	Saline	Rodríguez-Manzo, 2015
Rats (W), adult, sexually experienced, and sexually exhausted	0.001–0.1 mg/kg CNQX (AMPA/KA receptor antagonist)	Ip, 30 min before test	% of males MT, IO, EJ, resumed copulation. LT to IO, EJ; # of MT, IO; PEI. 30 min test	0.001 mg/kg: ↑ % of males EJ, # of MT, LT of EJ. 0.001 and 0.003 mg/kg: ↑ % of males MT, IO, resumed copulation, PEI	Saline	Rodríguez-Manzo, 2015
Rats (W), adult, sexually experienced, and sexually exhausted	0.003–0.3 mg/kg MPEP (mGluR5 antagonist)	Ip, 30 min before test	% of males MT, IO, EJ, resumed copulation. LT to IO, EJ; # of MT, IO; PEI. 30 min test	0.03 mg/kg: ↑ % of males MT, IO, EJ, resumed copulation; ↓ # of IO. 0.1 mg/kg: ↑% of males MT, IO, EJ	Saline	Rodríguez-Manzo, 2015
Japanese quail, adult, did not specify sexual experience, castrated, T-primed	100 μg LY367385 (mGluR1 antagonist)	3V, 30 min before testing	# of RCSM; contraction of cloacal sphincter muscles. 30 min test	↓ # of RCSM	Propylene glycol	Seredynski et al., 2015
Japanese quail, adult, did not specify sexual experience, castrated, T-primed	100 μg LY341495 (mGluR2/3 antagonist)	3V, 30 min before testing	# of RCSM; contraction of cloacal sphincter muscles. 30 min test	No changes	Propylene glycol	Seredynski et al., 2015
Japanese quail, adult, did not specify sexual experience, castrated, T-primed	100 μg MPEP (mGluR5 antagonist)	3V, 30 min before testing	# of RCSM; contraction of cloacal sphincter muscles. 30 min test	No changes	Propylene glycol	Seredynski et al., 2015
Rats (LE), adult, sexually naïve	2.5 μg MK801 (NMDA receptor antagonist)	mPOA, unilateral, 10 min before test	# of MT, IO, EJ; LT to MT, IO, EJ; PEI. # of MT and IO before 1st EJ. IO ratio total and before 1st EJ. IO interval. 30 min test	↓ # of MT and IO; ↓ IO ratio; ↑ LT to MT	ACSF	Vigdorchik et al., 2012
Rats (LE), adult, 1 sexual experiences that reached EJ	2.5 μg MK801 (NMDA receptor antagonist)	mPOA, unilateral, 10 min before test	# of MT, IO, EJ; LT to MT, IO, EJ; PEI. # of MT and IO before 1st EJ. IO ratio total and before 1st EJ. IO interval. 30 min test	↓ # of IO and EJ; ↓ IO ratio; ↑ LT to MT, IO, EJ	ACSF	Vigdorchik et al., 2012
Rats (LE), adult, 3 sexual experiences that reached EJ	2.5 μg MK801 (NMDA receptor antagonist)	mPOA, unilateral, 10 min before test.	# of MT, IO, EJ; LT to MT, IO, EJ; PEI. # of MT and IO before 1st EJ. IO ratio total and before 1st EJ. IO interval. 30 min test	No changes	ACSF	Vigdorchik et al., 2012
Rats (LE), adult, 3 sexual experiences that reached EJ, expose to inaccessible female for 7 consecutive days	2.5 μg MK801 (NMDA receptor antagonist)	mPOA, unilateral, 7 consecutive days, 10 min before exposing to female	# of MT, IO, EJ; LT to MT, IO, EJ; PEI. # of MT and IO before 1st EJ. IO ratio total and before 1st EJ. IO interval. 30 min test	↓ # of MT, IO EJ; ↓ IO ratio	ACSF	Vigdorchik et al., 2012
Rat (SD), sexually experienced	5 μg MK801 (NMDA receptor antagonist)	PVN, unilateral, 5 min before test	Non-contact erection scored with inaccessibly female. 40 min test. # of MT, IO, EJ. LT to MT, IO, EJ. PEI. 40 min test	↓ episodes of non-contact erections. ↑ LT to MT, IO, EJ. ↓ # of MT, IO	Ringer's solution	Melis et al., 2004
Rat (SD), sexually experienced	5 μg CNQX(AMPA receptor antagonist)	PVN, unilateral, 5 min before test	Non-contact erection scored with inaccessibly female. 40 min test. # of MT, IO, EJ. LT to MT, IO, EJ. PEI. 40 min test	↑ LT to MT, IO, EJ. ↓ # of MT, IO, EJ. ↑ PEI	Ringer's solution.	Melis et al., 2004
Rat (SD), sexually experienced	5 μg AP4 (mGluR4, 6, 7, 8 agonist)	PVN, unilateral, 5 min before test	Non-contact erection scored with inaccessibly female. 40 min test. # of MT, IO, EJ. LT to MT, IO, EJ. PEI. 40 min test	No changes	Ringer's solution	Melis et al., 2004
Rats (W), did not specify sexual experience, treated with PCA (induce EJ)	100 μg MMPIP (mGluR7 antagonist	It (L4-L5), 35 min before test	Seminal material weight onto paper or shaft of penis. 120 min measurement	↓ seminal material	DMSO	Masugi-Tokita et al., 2020
Rats (SD), 2 h sexual experience	0.07 and 0.1 mg/kg MK801 (NMDA receptor antagonist)	Ip. 3 days before test	LT to MT, IO, EJ. 30 min tests	↑ LT to MT	Saline	Fleming and Kucera, 1991

*mPOA, medial preoptic area; PVN, paraventricular nucleus; PEI, postejaculatory interval; #, number; LT, latency; MT, mount; IO, intromission; EJ, ejaculation; RCSM, rhythmic cloacal sphincter movements; SD, Sprague–Dawley; W, Wistar; LE, Long–Evans; T, testosterone; C57, C57BL/6N;MMPIP, NAM 6-(4-methoxyphenyl)-5-methyl-3-pyridinyl-4-isoxazolo[4,5-c]pyridin-4(5H)-one; BNST, bed nucleus of the stria terminalis; MeA, medial amygdala; icv, intracerebroventricularly; ip, intraperitoneally; im, intramuscularly; it, intrathecally*.

In tandem with the finding that 5 μg of MK801 administered to the PVN decreased male sexual behavior, Melis et al. (2004) explored how glutamatergic synapses may link oxytocinergic neurons from the limbic system. They then postulated how glutamatergic signaling is initiated at the electrophysiological level and proposed experiments using tetrodotoxins to discern if this signaling depended on the action potential. In recent studies, researchers have used advanced tools (anterograde and retrograde viral tracers, chemogenetics, and optogenetics) to manipulate the ventromedial hypothalamus (VMH)–anteroventral periventricular nucleus for female sexual behavior (Inoue et al., 2019) and posterior amygdala–medial preoptic nucleus circuit for male sexual behavior (Yamaguchi et al., 2020). If these advanced tools are coupled with experiments manipulating ionotropic GluRs in future studies, they would provide insights into how glutamate fits in neurotransmission that eventually leads to sexual behavior with high spatiotemporal resolution.

Supplementary to drug treatments, genetically engineered animal models may proffer important support to studying glutamate in sexual behavior. For example, mutant mice with reduced levels of GluN1 down to 5–10% of GluN1 expressed in wild type do not copulate with females (Mohn et al., 1999). Mohn et al. (1999) attributed the absence of male sexual behavior to social withdrawal as evidenced through their physical distant behavior with cage mates and the avoidance of interactions in the resident–intruder paradigm. A well-known caveat in studying mutant mice is that GluN1 is reduced in the whole organism (i.e., not limited to certain organs and cell types) since embryonic development. Therefore, this global effect renders it difficult to parse out GluN1's specific contributions to sexual behavior. To circumvent such limitations, we now have conditional knockout mice, such as the *Cre-lox* mice, to selectively delete genes in a specific organ and cell type (reviewed in Balthazart, 2020a).

All in all, several studies manipulated NMDA receptors with their antagonists MK801 and ketamine HCl. We can conclude that NMDA receptor is involved in the different consummatory phases of male sexual behavior including mounting, intromitting, and ejaculation, in addition to appetitive phases such as in sex-related vocalizations.

#### Manipulating Other Ionotropic Glutamate Receptors

Aside from NMDA receptor antagonists, other studies have used pharmacological agents targeting other GluRs. CNQX, an antagonist for AMPA and KA receptors, when administered intraperitoneally, increased the percentage of male Wistar rats that resumed male sexual behavior in sexually exhausted rats at 0.001 mg/kg concentration (Rodríguez-Manzo, 2015). Conversely, administering 5 μg of CNQX to the PVN of sexually experienced male Sprague–Dawley rats impaired several male sexual behavior parameters including increased latency to ejaculation and post-ejaculatory interval (Melis et al., 2004).

This disparity highlights the specificity of pharmacological effects depending on a variety of factors varying from the type of animal, route of administration, drug concentration, sexual behavior tested, brain regions targeted, type of antagonism, age at glutamate administration, and sexual status of the animal. This calls for future studies to discern the mechanisms underlying how ionotropic GluR antagonists affect male sexual behavior under differing variables. We also caution that the volume of drugs injected should not diffuse out of the intended brain region and that the damage from microinjections does not affect the intended behavior.

Another observation from the studies discussed so far pertains to the glutamate-related compounds that do not completely abolish sexual behavior. This raises queries on what auxiliary factors may be present that prevent the elimination of sexual behavior altogether. Potential studies to reveal this interaction could conduct experiments that co-administer other drugs with glutamate-related compounds.

#### Manipulating Metabotropic Glutamate Receptors

Regarding metabotropic GluRs, these comprise of GPCRs that signal more slowly relative to ionotropic GluRs and mostly function to inhibit postsynaptic sodium and calcium channels (Cachope and Pereda, 2020). Three studies have targeted mGluR5 using its antagonist, MPEP. In terms of rodent studies, intraperitoneal injection of 20 mg/kg MPEP to Long–Evans rats reduced male sexual behavior (e.g., increased latency to ejaculate, and post-ejaculatory interval) (Li et al., 2013). Another study discovered the opposite effect in sexually exhausted Wistar rats, where intraperitoneal injection of 0.03 mg/kg MPEP increased the percentage of males that resumed copulation (Rodríguez-Manzo, 2015). These discrepant effects between studies could arise from differences in the use of animal and strain, route of drug administration, drug concentration, and the sexual status of the animal. In terms of mGluR2/3, researchers observed a lack of effect in Long–Evans rats when they administered 1 and 3 mg/kg of the mGluR2/3 agonist LY379268 intraperitoneally (Li et al., 2013). This does not come as a surprise as mGluR2/3 do not express in the mPOA (Li et al., 2013).

One study examined mGluR7 with a 20-mg/kg intraperitoneal injection of its agonist, AMN082 to Long–Evans rats (Li et al., 2013). This treatment decreased male sexual behavior (increase in latency to ejaculate and post-ejaculatory intervals). The use of AMN082 to study behavior has been questioned, as AMN082 has been shown to induce locomotor deficits that may confound the intended behavior (Masugi-Tokita et al., 2020); however, further experiments by Li et al. (2013) failed to reveal sedation and locomotor activity changes. AP4 provides another mGluR7 agonist to test for male sexual behavior. When 5 μg of AP4 was administered to the PVN of Sprague–Dawley rats, no changes in male sexual behavior occurred (Melis et al., 2004). It should be noted that in interpreting these results, one has to consider the non-specific effects of AP4, as AP4 can also act as agonists for mGluR4, 6, and 8. With regard to mGluR7 antagonists, when 1.25 μg of MMPIP was administered to the bed nucleus of the stria terminalis, this treatment led to an increase in the percentage of male C57BL/6J mice that mounted (Masugi-Tokita et al., 2016). However, as noted by the authors, the mounting geared toward intruder conspecific males may be related to aggression rather than sexual behavior. In a relevant study using male Wistar rats treated with PCA to induce ejaculation, 100 μg of MMPIP administered intrathecally decreased the amount of seminal material ejaculated (Masugi-Tokita et al., 2020). In addition to MMPIP treatment, Masugi-Tokita et al. (2020) studied mice deficient in mGluR7 and found them to exhibit deficits in ejaculation, which they found were not due to impairments in sexual motivation or erection abilities. In summary, these studies targeted mGluR5 with their antagonist, and R mGluR7 with their agonist and antagonists, and taken together, the results of these studies support the conclusion that both of these mGluRs impart significant influence on rodent male sexual behavior.

### Glutamate Transporters and Rodent Male Sexual Behavior

Glutamate transporters carry glutamate across a membrane either from the synaptic cleft [excitatory amino acid transporter (EAAT)] or from the cytoplasm into synaptic vesicles (VGLUT) (reviewed in Tzingounis and Wadiche, 2007). While VGLUT is neuron-specific, some EAATs express in the astrocytes including EAAT1 (also known as GLAST) and EAAT2 (also known as GLT1) (reviewed in Blanco-Suárez et al., 2017). Glutamate that is released from neurons transports into glial cells to convert to its non-toxic form, glutamine, thereby rendering this mechanism crucial to minimize neuron death (reviewed in Haroon et al., 2017).

The astrocytes that facilitate this glutamine–glutamate cycle, together with pre- and post-synaptic membrane, form the tripartite synapse (reviewed in Haroon et al., 2017). In this tripartite synapse, astrocytes themselves drive neurotransmission through their ability to release calcium quasi to neurons, in addition to detecting and responding to signals from neurons to mediate synaptic plasticity (reviewed in Blanco-Suárez et al., 2017). In concordance with this view, emerging studies demonstrated glutamate transporters to exhibit roles in synapse transmission (reviewed in Gonçalves-Ribeiro et al., 2019). This occurs through their competition with GluRs for glutamate as well as regulating ion concentrations imperative for GluRs (reviewed in Gonçalves-Ribeiro et al., 2019). To this end, glutamate transporters facilitate synaptic plasticity through LTP that induces long-lasting neurotransmission (reviewed in Gonçalves-Ribeiro et al., 2019).

Recent studies have begun to explore the role of glutamate transporters on behavior. For instance, reduced astrocytic EAAT2 correlated with chronic social defeat stress in a rat depression model (Rappeneau et al., 2016). Another example examined mice deficient in astrocytic EAAT2 and uncovered that these mice exhibited less anxiety and depression-related behavior (Jia et al., 2020). Relating to sexual behavior, researchers tested the effects of glutamate transporter inhibitors on the male sexual behavior of Long–Evans rats (Dominguez et al., 2006). The inhibitors they used were a mixture of 250 μM L-trans-2,4-PDC (EAAT inhibitor) and 250 μM Chicago sky blue (VGLUT inhibitor), which was reverse-dialyzed into the mPOA. This treatment increased their number of ejaculations and decreased their latency to ejaculate. Interestingly, the inhibitors did not affect ejaculation immediately, and the authors attributed this to the limited volume injected. On the note of neuron populations, these drug actions may first act on the targeted brain regions and, in turn, affect their downstream projections to affect sexual behavior. Considering this projection-based paradigm, future studies that would map the sexual behavior projections in other brain regions to and from the target site would help in deducing the spatiotemporal effects of glutamate-related drugs.

Given that these inhibitors not only target neuron-specific EAAT, it remains unknown whether some of these effects on sexual behavior are governed through effects from glial EAAT. EAATs sensitize to sex steroid hormone cycles; for example, estrogen and progesterone priming increased glial fibrillary acidic protein immunoexpression (astrocytic marker) in the MeA (Martinez et al., 2006). Moreover, during development, males harbored more microglia in the POA compared to females and were deemed critical for exhibiting male sexual behavior during adulthood (Lenz et al., 2018).

In terms of glia affecting sexual behavior, Grosjean et al. (2008) demonstrated in male *Drosophila melanogaster* a glial amino-acid transporter regulated courtship and copulatory behavior toward other males. A more recent study showed that in male *Caenorhabditis elegans*, transdifferentiation occurs in glial cells that convert to neurons important for copulation (Molina-García et al., 2018). There abide a paucity of knowledge on how glia regulates sexual behavior, and given their critical role in regulating glutamate, future experiments can help elucidate the specific role of glia on glutamatergic signaling in sexual behavior.

### Potential Role of Glutamate in Steroid-Independent Male Sexual Behavior

While male sexual behavior can be reinstated in castrated rodents with the administration of testosterone, different proportions of males resumed ejaculation at different testosterone concentrations and periods in golden hamsters and Long–Evans rats, suggesting steroid-independent factors at play (Tiefer and Johnson, 1973; Harding and Velotta, 2011). It would be of interest to investigate how glutamate and their receptors and transporters may factor in this variation. In support of this, a microarray study uncovered several differentially expressed genes that relate to glutamate between hybrid B6D2F1 male mice that exhibited steroid-independent sexual behavior (maters) and those that did not (non-maters) (Park et al., 2010). The maters, relative to the non-maters, displayed up-regulated mRNA of several genes in the mPOA such as microtubule-associated protein tau and *App*, as well as down-regulated mRNAs such as phosphatase and tension homolog, inositol(myo)-1(or 4)-monophosphatase 1, voltage-gated type I alpha sodium channel, and *Sod* (Park et al., 2010; Bharadwaj et al., 2013). Further studies identified increased immunoexpression of synaptic proteins, synaptophysin, and spinophilin, as well as the growth of dendritic spine density (Bharadwaj et al., 2013). From the microarray study, a myriad of differentially regulated genes may suggest the participation of glutamatergic signaling in steroid-independent sexual behavior, and we discuss two of these here: *App* and *Bdnf*.

The transmembrane protein *App* acts on axon growth, axon guidance, and synaptic function, and plays a significant role in learning (reviewed in Müller et al., 2017). Park et al. (2010) further validated *App* up-regulation in maters using qPCR, and transgenic mice with overexpressed *App* exhibited post-castration ejaculation at least 20 weeks in 20% of them (Park et al., 2010). Apropos to glutamatergic signaling, some evidence suggests that *App* participates in this system. For example, in the *Drosophila* model, *App* modulated sleep through the glutamate recycling components: *Drosophila* EAAT1 and glutamine synthetase (Farca Luna et al., 2017). In tandem with that, *App* regulated LTP through stimulating the trafficking of AMPA and NMDA receptors to the extra-synaptic cell surface (Mockett et al., 2019). Moreover, APP tuned glutamate release by acting on synaptic vesicle protein and neurotransmitter release machinery (Yao et al., 2019).

Next, *Bdnf*, a critical factor in neuron survival (Harward et al., 2016), displayed up-regulation in maters; however, supplementary validation experiments still awaits (Park et al., 2010). With regard to glutamatergic signaling, *Bdnf* release depends on the NMDA receptor in stimulated dendritic spines (Harward et al., 2016). Corresponding to this *Bdnf* –glutamate interaction, the modulation of glutamate release likewise depended on *Bdnf* secretion in oligodendrocytes (Jang et al., 2019). Furthermore, the effects of methyl CpG binding protein 2 alteration toward glutamatergic neurons are conditioned on *Bdnf* expression (Sampathkumar et al., 2016). Although this work appears tangential to supporting our postulation, it demonstrates the lacuna that exists for fully grasping the detailed mechanisms of glutamatergic signaling in sexual behavior.

We acknowledge that these observations corroborate only by correlation and only peripheral to support glutamate's role in steroid-independent sexual behavior. Understanding the deeper transcriptome and proteome in steroid-independent sexual behavior models focusing on glutamate (plus their receptors and transporters), followed by manipulation methods, would help us further elucidate this narrative. Other lines of research to suggest that glutamate may play a role in gonadal steroid-independent sexual behavior pertains to studies that linked glutamate and enhanced male sexual behavior in sexually experienced male rodents relative to those that were sexually naive, as well as studies associating glutamate in the “studs vs. duds” phenomenon.

### Sexual Experience

Sexual experience tenders tremendous changes in neuroplasticity, which include increases in neurogenesis and dendritic spine density, in altering electrophysiology properties, as well as changes in signaling (e.g., BDNF, nitric oxide synthase) and gene expression (e.g., immediate early genes) (reviewed in Herrera-Morales et al., 2019). At the behavioral level, sexual experience decreases the latency to ejaculate in male Wistar rats (Garcia-Martinez et al., 2010) and improves memory in middle-aged Sprague–Dawley rats (Glasper and Gould, 2013). Sexually experienced male rodents retain components of male sexual behavior longer than sexually naive male rodents after castration, suggesting gonadal steroid-independent mechanisms at work (reviewed in Park and Rissman, 2011). Pre-castration sexual experience prolonged retention of sexual behavior after castration in golden hamsters (Lisk and Heimann, 1980). Although not necessary, pre-castration sexual experience led to a higher proportion of male hybrid B6D2F1 mice to demonstrate steroid-independent male sexual behavior after castration relative to their sexually naïve counterparts (Manning and Thompson, 1976). We now review a few studies that addressed the interesting relationship between glutamate and the changes associated with sexual experience.

We discussed before that mating increased GluN1 phosphorylation in the mPOA of Sprague–Dawley rats (Dominguez et al., 2007). It turns out chronic changes occur to NMDA receptors in response to additional sexual experience (Pitchers et al., 2012). These include (1) an increased GluN1 surface and intracellular expression 1 day after mating, (2) increased intracellular GluA2 1 week after, and (3) increased surface GluA2 expression 1 month after. Consistent with these results, Western blotting from sexually experienced male C57Bl/6J mice revealed an increase in GluN1 (Jean et al., 2017). In the case of manipulating GluRs, one study directly compared the effects between sexually naïve and sexually experienced rodents (Vigdorchik et al., 2012). Male sexual behavior diminished (e.g., decreased intromission ratio) in Sprague–Dawley rats administered with 2.5 μg of MK801 to the mPOA that were either sexually naïve or had one sexual experience; however, no changes in male sexual behavior took place by similar treatments in sexually experienced rats (those that had three sexual experiences).

Most rodent studies manipulating GluRs in male sex behavior studies have only utilized sexually experienced rodents. In some cases, researchers did not specify prior sexual experience of the subjects, thus rendering it difficult to parse out how much of a factor sexual experience may play in those studies (e.g., Giuliano et al., 1996; Brudzynski and Pniak, 2002). Reporting details of the sexual experience, such as the number and duration of each sexual encounter and quantification of the sexual behavior, would benefit future studies in disentangling the role of glutamate in all stages of sexual behavior.

Sex steroid hormones can partially explain the effects of sexual experience. For example, one study showed a larger plasma testosterone boost during sexual behavior in sexually experienced male Wistar rats relative to controls (Bonilla-Jaime et al., 2006). Furthermore, another study demonstrated an increase in the numbers of androgen receptor-positive cells in the mPOA in sexually experienced C57BL/6 male mice relative to controls (Swaney et al., 2012). Although any behavioral and neuroplasticity changes due to sexual experiences involving glutamate mechanisms may interact with sex steroid hormones, future research could test the direct causality of testosterone's participation in glutamate-mediated sexual experience changes along with potential steroid-independent mechanisms.

### Studs vs. Duds

The second line of research that suggests a role of glutamate in gonadal steroid-independent sexual behavior also involves individual differences, specifically the phenomenon known as “studs vs. duds.” This phenomenon describes gonad-intact male rodents in which subgroups either demonstrate low or high levels of male sexual behavior during sexual behavior tests (Damassa et al., 1977). Heterogenous phenotypes of male sex behavior manifested also in Wistar rats, in which different groups of rats were categorized into separate endophenotypes as either sluggish, normal, or rapid ejaculators based on the ejaculation frequencies across five sexual behavior tests (Pattij et al., 2005; Olivier et al., 2011). The “studs vs. duds” phenomenon does not depend on sex steroid hormones as the endophenotypes do not correlate with levels of testosterone, progesterone, or estradiol (Kohlert and Block, 1996; De Gasperín-Estrada et al., 2008). In support of these results, chronic treatment of estradiol or testosterone for 12 weeks did not boost sexual behavior in sexually sluggish male Wistar rats (Antonio-Cabrera and Paredes, 2012).

Several studies have investigated the potential role of glutamate in explaining these individual differences. One study explored the role of ionotropic GluR agonists in sexually sluggish and good copulators (Drago and Busǎ, 1997). Ionotropic GluRs are named according to the selective agonists that can activate them (Tzingounis and Wadiche, 2007). Accordingly, NMDA, AMPA, and KA are agonists for NMDA, AMPA, and KA receptors, respectively (Tzingounis and Wadiche, 2007). Intraperitoneal injections of KA to sexually sluggish Wistar rats increased male sexual behavior (e.g., decreased latency to intromit) at 1 and 2.5 mg/kg concentration (Drago and Busǎ, 1997). Meanwhile, the highest concentration administered (5 mg/kg) hindered male sexual behavior instead (e.g., increased latency to ejaculate) (Drago and Busǎ, 1997). This effect was replicated in good copulators at the 5-mg/kg concentration but failed to exert changes at the lower 1- and 2.5-mg/kg KA concentration (Drago and Busǎ, 1997). These sexual endophenotypes exhibit neurobiology variations where the rapid ejaculators harbored more FOS-positive neurons in the supraoptic nucleus of the hypothalamus than the sexually sluggish rats (Pattij et al., 2005). Future experiments can look at effects from other glutamate-related compounds, as well as inherent glutamatergic differences among these endophenotypes.

While several studies have investigated this endophenotype, Trejo-Sánchez et al. (2020) call these sexual endophenotypes into question due to the lack of internal consistencies at various levels of organization. Specifically, although these sexual endophenotypes exhibited differences in ejaculation frequencies and latencies, no differences appeared when they compared the latencies to mounting and intromission as well as the number of mounting and intromission in these groups. Furthermore, they detected no expression differences in a panel of genes between these sexual endophenotypes in the amygdala, olfactory bulb, mPOA, and VMH: estrogen receptor, progesterone receptor, androgen receptor, aromatase, and DNA methyltransferase. Therefore, further work awaits to clarify these discrepancies between studies.

## Rodent Female Sexual Behavior

Sex differences exist in glutamate levels and expression of glutamate receptor and transporters (reviewed in Giacometti and Barker, 2020). Therefore, the landscape of glutamatergic signaling would likely differ for female sexual behavior to males. Their repertoire of sexual behavior encompasses attractivity, proceptivity, and receptivity. Attractivity and proceptivity behaviors aim to solicit male mounting such as hopping, darting, and ear wiggling (reviewed in Micevych and Meisel, 2017; Rudzinskas et al., 2019). In response to male mounting, the female displays receptivity through arching their back, a behavior termed lordosis (reviewed in Micevych and Meisel, 2017). Different parameters to measure lordosis include the lordosis quotient, which scholars defined as the number of lordosis reflexes in response to the number of male mounts (reviewed in Micevych and Meisel, 2017).

Generally, female sexual behavior governs through sex steroid hormones secreted by the ovaries: estradiol and progesterone (reviewed in Micevych and Meisel, 2017). Estrogen levels peak during the proestrous phase followed by a peak in progesterone levels in estrous, and they act through their cognate receptors, estrogen, and progesterone receptors (reviewed in Rudzinskas et al., 2019). With respect to the neural circuitry for female sexual behavior, decades of research has mapped this extensively (reviewed in Tetel and Pfaff, 2010; Tobiansky et al., 2013, 2016; Micevych and Meisel, 2017). Among other brain regions, these include the ventrolateral portion of the VMH, the basal forebrain, arcuate nucleus, mPOA, VTA, and NAc.

It should be noted that individual differences are an important characteristic found in female sexual behavior. For instance, in ovariectomized female Damaraland mole rats, over 87% of the females displayed lordosis, with no differences in the frequencies of solicitations when compared to the gonad-intact females in 12 sexual behavior tests (Carter et al., 2014).

### Glutamate and Rodent Female Sexual Behavior

Studies have demonstrated a strong relationship between glutamate and female sexual behavior; in general, glutamate is released during female sexual behavior in the NAc and VMH. During vaginocervical stimulation, FOS-positive glutamatergic neurons increased, and glutamate is released in the VMH of Long–Evans rats (Georgescu et al., 2009, 2014). A more recent study demonstrated that in Syrian hamsters, glutamatergic neurons (CaMKIIa+) in the mPFC that projected to the NAc also became FOS-positive during sexual testing (Moore et al., 2019). Furthermore, they used biosensors to find glutamate release in NAc, and the release both preceded and occurred during intromission (Moore et al., 2019). Additional work to build upon these studies could study glutamate release in other brain regions pertinent to female sexual behavior, in conjunction with how glutamate release synchronizes with different phases of sexual motivation. A minor caveat to these studies refers to the use of FOS immunohistochemistry for neuron activation. The utility of FOS to indicate neuron activation depends on the release of calcium, but this may not always occur during neuron firing (Hitora-Imamura et al., 2015).

A way around this would entail deploying direct electrophysiological recordings of the neurons *in vivo*. Coupled with microdialysis and biosensors to measure glutamate release, the recent development of genetically encoded glutamate indicators enables the visualization of glutamate release *in vivo* such as the intensity-based glutamate-sensing fluorescent reporter (Marvin et al., 2018). Marvin et al. (2018) transduced cells in the mice somatosensory cortex and ferret visual cortex with virus expressing these reporters and successfully detected glutamate release from neuropil, cell bodies, and dendritic spines in orientation-selective neurons in response to grating stimuli (Marvin et al., 2018).

Two studies administered glutamate directly to the VMH. In one study, female rats (strain unspecified) declined in lordosis reflex intensity in response to manual stimulation (placing pressure on the animal's flanks) at all concentrations used above 1.0 mM (Kow et al., 1985). Disparate to that, lordosis did not alter when another study gave the same concentration to female Long–Evans rats (Georgescu and Pfaus, 2006). That said, they did find decreases in the number of hops and darts. The difference in methods inducing lordosis probably resulted in these inconsistencies. Kow et al. (1985) employed manual flank stimulation for sexual behavior testing, which would lack the full sensory stimulation from actual male rats. We summarized these glutamate application studies in female animals, as well as subsequent studies reviewed in Table 2.

**Table 2:** Effects of glutamate receptor antagonists and agonists and glutamate on female sexual behavior.

**Female species of strain**	**Drug and dose**	**Administration**	**Sexual behavior assessed**	**Sexual behavior changed**	**Comparison sample**	**References**
Rats (LE), adult, OVX, HP, sexually experienced	200 ng MK-801 (NMDA receptor antagonist)	VTA, bilateral, 40 min before test	Paced mating: LQ, aggression quotient, pacing exits. 15 min test	↑ LQ	Saline	Frye et al., 2020
Rats (SD), adult, OVX, HP, sexually experienced	15 nmol AP7 (NMDA receptor antagonist) then another 10 nmol AP7	3V, 1st 3 h before test, then 2nd 1.5 h before test	LQ to male mount, LR magnitude. 15 min test	↓ LQ	Saline	Gargiulo et al., 1992
Rats (SD), adult, OVX, HP, sexually experienced	15–30 nmol DNQX (AMPA and KA receptor antagonist) then another 10–20 nmol DNQX	3V, 1st 3 h before test, then 2nd 1.5 h before test	LQ to male mount, LR magnitude. 15 min test	No changes	Saline	Gargiulo et al., 1992
Rats (LE), adult, OVX, HP, sexually experienced	3.3, 10, 100 mmol glutamate	VMH, bilateral, did not specify infusion time	# of SOL, hops and darts, pacing, rejection responses; LQ to male mount, LR magnitude. 30 min test	↓ # of hops and darts	Saline	Georgescu and Pfaus, 2006
Rats (LE), adult, OVX, HP, sexually experienced	0.3, 1, 2 mmol AMPA	VMH, bilateral, did not specify infusion time	# of SOL, hops, darts, pacing, rejection response; LQ to male mount; LR magnitude. 30 min test	All concentrations: ↓ SOL, hop and dart; ↓ LQ, LR magnitude; 0.33 mmol: ↑ rejection response	Saline	Georgescu and Pfaus, 2006
Rats (LE), adult, OVX, HP, sexually experienced	1, 3.39, 6.8 nmol NMDA	VMH, bilateral, did not specify infusion time	# of SOL, hops, darts, pacing, rejection response; LQ to male mount; LR magnitude. 30 min test	3.39 nmol: ↑ pacing and rejection response; ↓ LQ	Saline	Georgescu and Pfaus, 2006
Rats (LE), adult, OVX, HP, sexually experienced	0.469, 0.938, 1.17 mmol KA	VMH, bilateral, did not specify infusion time	# of SOL, hops, darts, pacing, rejection response; LQ to male mount; LR magnitude. 30 min test	All concentration: ↓ SOL, LQ; ↑ pacing. 1.17 mmol: ↓ hop and dart	Saline	Georgescu and Pfaus, 2006
Rats (LE), adult, OVX, HP, received 1 VCS, sexually experienced	19.8 mmol DNQX (AMPA and KA receptor antagonist)	VMH, bilateral, right before VCS	Paced mating: # of SOL, hops, dart, pacing, defensive response; LR magnitude, LQ. 30 min test	No changes	Saline	Georgescu et al., 2012
Rats (LE), adult, OVX, HP, received 50 VCS, sexually experienced	19.8 mmol DNQX (AMPA and KA receptor antagonist)	VMH, bilateral, right before VCS	Paced mating: # of SOL, hops, dart, pacing, defensive response; LR magnitude, LQ. 30 min test	↑ # of SOL, hops, dart; LR magnitude, LQ	Saline	Georgescu et al., 2012
Rats (LE), 2.5 months old, OVX, HP OVX, did not specify sexual experience	40 mg/kg NMDA	ip, 10 min before test	LQ, 20 min test	↑ LQ	Before NMDA injection	Hsu et al., 1993
Rats (LE), 2.5 months old, OVX, HP, treated with 4 mg/g monosodium glutamate at P1 and P3, did not specify sexual experience	40 mg/kg NMDA	ip, 10 min before test	LQ, 20 min test	No changes	Before NMDA injection	Hsu et al., 1993
Rats (strain not specified), adult, OVX, HP, 5 sexually experience	2 mmol AMPA	Ventrolateral VMH, bilateral, on test days 2, 3, and 4, did not specify infusion time	# of SOL, hop, dart, defensive behavior; LR magnitude. 30 min test for 5 days	↑ # of LR with a magnitude of 3 on test 5	Saline	Jones et al., 2015
Rats (SD), adult, OVX, HP, did not specify sexual experience	50 ng AP5 (NMDA receptor antagonist)	MBH, bilateral, 10 min before test.	LQ, vocalization. Duration of 3–10 male mounts	No changes	Same rat, before drug infusion	McCarthy et al., 1991
Rats (SD), adult, OVX, HP, did not specify sexual experience	50 ng AP5 (NMDA receptor antagonist)	POA, bilateral, 10 min before test	LQ, vocalization. Duration of 3–10 male mounts	↓ LQ	Same rat, before drug infusion	McCarthy et al., 1991
Rats (SD), adult, OVX, HP, did not specify sexual experience	20 ng NMDA	MBH, bilateral, 10 min before test	LQ, vocalization. Duration of 3–10 male MT	↓ LQ; ↑ vocalization	Same rat, before drug infusion	McCarthy et al., 1991
Rats (SD), adult, OVX, HP, did not specify sexual experience	20 ng NMDA	POA, bilateral, 10 min before test	LQ, vocalization. Duration of 3–10 male MT	No changes	Same rat, before drug infusion	McCarthy et al., 1991
Hamsters (golden), adult, OVX, HP, sexually experienced	0.375 μg KA	Lateral septal area, bilateral, at time of brain surgery	Latency to 1st lordosis, duration of a longest single bout of lordosis; duration of total lordosis. 10 min test	↓ duration of a longest single bout of lordosis and duration of total lordosis; ↑ latency to 1st lordosis	Saline	Nance and Myatt, 1987
Rats (W), adult, OVX, HP, sexually naïve	50 μM kynurenic acid (NMDA, AMPA, KA receptor antagonist)	NAc shell, unilateral. 10 min before + time until reaching dopamine baseline	Preference for male-soiled bedding: digging and investigation time. 40 min test	↓ investigation time	ACSF	Sánchez-Catalán et al., 2017
Rats (SD), OVX, HP, did not specify sexual experience	0.5 μg KA	Lateral septum, bilateral, 6 weeks before test	LQ. Until 10–15 MT occurred	↓ LQ	Saline-ascorbic acid	Nance, 1983
Rats (SD), HP, did not specify sexual experience	1 μg KA	Lateral septum, bilateral, 60 days before test	LQ. Until 10–15 MT occurred	↓ LQ	Saline	King and Nance, 1986
Rats (SD), HP, did not specify sexual experience	1 μg KA	Hippocampus, bilateral, 60 days before test	LQ. Until 10–15 MT occurred	No changes	Saline	King and Nance, 1986
Rats (SD), HP, did not specify sexual experience	2 μg KA	Medial septum, unilateral, 60 days before test	LQ. Until 10–15 MT occurred	No changes	Saline	King and Nance, 1986
Rats (SD), daily injected with TP over 21 days, sexually experienced	1 μg KA	Lateral septum, bilateral, 80 days before test	# of MT; LT of MT. 15 min	No changes	Saline	King and Nance, 1986
Rats (SD), daily injected with TP over 21 days, sexually experienced	1 μg KA	Hippocampus, bilateral, 80 days before test	# of MT; LT of MT. 15 min	↑ # of MT	Saline	King and Nance, 1986
Rats (SD), daily injected with TP over 21 days, sexually experienced	2 μg KA	Medial septum, unilateral, 80 days before test	# of MT; LT of MT. 15 min	No changes	Saline	King and Nance, 1986
Rats (SD), adult, OVX, HP, did not specify sexual experience	0.05–0.4 mg/kg MK801 (NMDA receptor antagonist)	Sc. 30–50 min before test	LQ, lordosis score. Presence of darting and ear wiggling. Duration of 10 male MT	0.05, 0.1, 0.2, 0.4 mg/kg: ↓ LQ, lordosis score, no ear wiggling.0.1, 0.2, 0.4 mg/kg: no darting	Saline	Fleischmann et al., 1991
Rats (SD), adult, OVX, HP, did not specify sexual experience	30 mg/kg Dextrorphan (NMDA receptor antagonist)	Did not specify injection method. 60 min before test	LQ, lordosis score. Presence of darting and ear wiggling. 30 min test	↓ LQ and lordosis score	Did not specify control	Fleischmann et al., 1991
Rats (SD), adult, OVX, HP, did not specify sexual experience	0.5 mg/kg MK801 (NMDA receptor antagonist)	Sc. 48 h before test	LQ, lordosis score. Presence of darting and ear wiggling. Duration of 10 male MT	↓ LQ, lordosis score, no darting and ear wiggling	Saline	Fleischmann et al., 1991
Rats (LE), adult, OVX, HP, adrenalectomy, did not specify sexual experience	200 ng MK801 (NMDA receptor antagonist)	VTA, bilateral, 30 min before test	Paced mating: LQ, defensive aggression, pacing of sexual contact, % mating	↑ % mating, LQ	Saline	Frye and Paris, 2011
Rats (strain not specified), adult, OVX, HP, did not specify sexual experience	1, 3.3, 10, 100 mM glutamate	VMH, bilateral, immediately before test	LR intensity from manual stimulation. 60 min test	All concentration except 1.0 mM: ↓ LR up to 20 min	Same rat, 20 min before progesterone HP	Kow et al., 1985
Rats (strain not specified), adult, OVX, HP, did not specify sexual experience	0.5 μg KA	VMH, bilateral, immediately before test	LR intensity from manual stimulation. 60 min test	↓ LR	Same rat, 20 min before progesterone HP	Kow et al., 1985

*OVX, ovariectomized; HP, hormonally primed; LE, Long–Evans; #, number; LQ, lordosis quotient; LR, lordosis reflex; SOL, solicitations; SD, Sprague–Dawley; W, Wistar; AP7, 2-amino-7-phosphonoheptanoic acid; VCS, vaginocervical stimulation; VMH, ventromedial hypothalamus; POA, preoptic area; MBH, mediobasal hypothalamus; 3V, third ventricle; Sc, subcutaneous; ip, intraperitoneal*.

An important control to experiments administering glutamate-related compounds requires determining if the compound diffuses from the injection site to other unintended brain regions. To examine this, studies could monitor the response time of the sexual behavior from the time of administration, whether the concentration of the compound rises in neighboring brain regions, and administer the compound to multiple target sites to resolve which sites deliver the most pronounced effects. As can be seen from studies targeting different brain regions for glutamate measurement and administering glutamate, we conclude that glutamate partakes in female sexual behavior through glutamate release during sexual repertoire and direct action to manipulate these repertoires.

### Glutamate Receptors and Rodent Female Sexual Behavior

#### NMDA Receptor Antagonists

Several studies examined the effects of ionotropic GluR antagonists in female sexual behavior. Administration of the NMDA receptor antagonist, MK801, to the VTA (200 ng) of Long–Evans rats led to an increase in the lordosis quotient (Frye and Paris, 2011). This contrasts an earlier study that also administered MK801 but discovered a decreased lordosis quotient (Fleischmann et al., 1991). This disparity could arise due to the much higher dose of MK801 administered (0.05–0.4 mg/kg), a different rat model investigated (Sprague–Dawley), and the administration method (subcutaneous). A more compelling reason ascribes to the confounding behaviors exhibited in these mice, where they revealed changes in movements such as jumping, rearing, and circling (Fleischmann et al., 1991). On top of that, the two studies employed different sexual behavior testing paradigms. Frye and Paris (2011) allowed for paced mating, which enables the female to dictate sexual behavior through a partition that permits the female, but not the male, to cross over to another section of the testing arena due to differences in body size (Frye and Paris, 2011). The difference extends to molecular changes as paced mating is associated with increased neurogenesis, opioid release, and opioid receptor gene expression in the female rodents relative to non-paced mating (Ventura-Aquino and Paredes, 2020).

Other studies investigated other NMDA receptor antagonists (AP5, AP7, dextrorphan, and kynurenic acid), and administration of these NMDA receptor antagonists diminished female sexual behavior in rats (Fleischmann et al., 1991; McCarthy et al., 1991; Gargiulo et al., 1992; Sánchez-Catalán et al., 2017). In support of this, delivery of 50 μM kynurenic acid to the NAc decreased the investigation time of male-soiled bedding by the female Wistar rats (Sánchez-Catalán et al., 2017); however, this effect cannot ascribe directly to the NMDA receptor, as kynurenic acid also targets the glycine binding sites of AMPA and KA receptors (reviewed in Tzingounis and Wadiche, 2007). One study did find a lack of change in female sexual behavior to one NMDA receptor antagonist when the researchers administered 50 ng of AP5 to the mediobasal hypothalamus in female Sprague–Dawley rats (McCarthy et al., 1991). This probably accounted for the fact that the brain region targeted did not exert significant influence on sexual behavior, at least vis-à-vis antagonistic mechanisms of NMDA receptors. None of the other glutamate studies in sexual behavior in both females and males targeted the mediobasal hypothalamus.

Given the importance of glutamate in learning and memory (reviewed in Giacometti and Barker, 2020), it troubles us that this remains largely neglected in studies investigating the role of glutamate in sexual behavior. Fleischmann et al. (1991) noted this concern at a time when where only a paucity of data on the incentive reward properties of sexual behavior existed. Reward distinguishes into different phases from cue detection, anticipation, approaching, waiting, consummatory phase, post-consummatory phase, and motivation for an additional reward (Wang et al., 2017). Recent studies have begun to study this with high spatiotemporal resolution. A fiber photometry study found that serotoninergic neurons in the dorsal raphe nucleus encoded signals for the anticipatory and consummatory phases of the rewarding aspects of sexual behavior (Li et al., 2016). Future glutamate in sexual behavior studies should take learning and memory into account and elucidate the distinct glutamatergic signaling involved in the different aspects of sexual behavior from reward to the motor and behavioral components. In essence, from these NMDA receptor antagonist studies deploying a wide range of compounds, we conclude that NMDA receptor participates in female sexual behavior.

#### Other Ionotropic Receptor Antagonists

In conjunction with other ionotropic GluR antagonists, two studies applied DNQX, a competitive antagonist for AMPA and KA receptors. First, researchers dispensed 15–30 nmol DNQX to the third ventricle of Sprague–Dawley rats 3 h before testing for female sexual behavior and then another 10–20 nmol DNQX 90 min before testing; neither treatment affected female sexual behavior (Gargiulo et al., 1992). A second study administered 19.8 nmol DNQX to the VMH of Long–Evans rats that received one vaginocervical stimulation prior to paced mating, and this treatment also did not affect female sexual behavior (Georgescu et al., 2012). Normally, repeated vaginocervical stimulation leads to estrous termination and diminished sexual behavior; thus, their lordosis quotient and lordosis magnitude decreases (Georgescu et al., 2012). Interestingly, after female rats received vaginocervical stimulation 50 times, DNQX treatment reversed these effects (Georgescu et al., 2012).

These, and all of the studies discussed so far, accentuate how multiple factors require considerations for interpreting the results. This calls for the importance of reporting and standardizing procedures as well as experimental variables to enable valid comparisons. To name a few, these could relate to the experimenters that handle these animals (Sorge et al., 2014; Gouveia and Hurst, 2017), drug administration paradigm, sexual stimuli, sexual behavior testing, sexual behavior measured, light/dark cycle, food and water, housing enrichments, social conditions, surgical history, and conditions during the perinatal period.

#### Ionotropic Glutamate Receptor Agonists

In Long–Evans female rats, infusion of ionotropic GluR agonists AMPA or KA into the VMH at varying concentrations decreased the lordosis quotient at most of the concentrations tested (Georgescu and Pfaus, 2006). One study found that 20 ng of NMDA delivered to the mediobasal hypothalamus similarly diminished the lordosis quotient in Sprague–Dawley rats (McCarthy et al., 1991). When the same study administered this to the POA, there was no such effect. In contrast to these studies, when researchers injected 40 mg/kg NMDA intraperitoneally into Long–Evans rats, the lordosis quotient increased (Hsu et al., 1993). These studies again emphasize how varying effects of sexual behavior can result when the administration route differs. The inconsistent units used to report drug concentrations across studies also make it more difficult to swiftly compare the concentrations used between different studies.

Several studies have investigated ionotropic GluR agonists. KA (0.375 μg) injected to the lateral septal area reduced lordosis in female golden hamsters (Nance and Myatt, 1987). Researchers identified the same effect when they applied 0.5 μg of KA to the VMH of rats (strain unspecified); this was from manual stimulation, which was the researcher manually placing pressure on the animal's flanks (Kow et al., 1985). These negative effects on lordosis concurred with another study that applied 0.469–1.17 mmol KA, also to the VMH in Long–Evans rats Georgescu and Pfaus (2006). The same study also administered 0.3–2 mmol of AMPA into the VMH and found the same negative effects on lordosis. This contrasted with another study that enhanced lordosis after administering 2 mmol AMPA to the ventrolateral VMH on each of days 2, 3, and 4 out of the total 5 days of daily mating (strain not specified) (Jones and Pfaus, 2014). The differences in the testing paradigm most likely contributed to these opposing effects.

In dealing with ionotropic GluR agonists, it should be noted that certain concentrations may be neurotoxic. For example, the KA studies reported their dose to kill off neurons in the region (Nance and Myatt, 1987; Hsu et al., 1993). Scholars should therefore be cautious when interpreting sexual behavior studies that involve certain ionotropic GluR agonists, as the observation may result from the destruction of the neurons in the regions in lieu of *bona fide* glutamate signaling. Thus, future studies would profit from supplementary drug dose and histological data that evince the targeted regions remain undamaged. The other two KA studies did specify that the concentrations they used were 10 times smaller than the concentrations known to induce lesions (Georgescu and Pfaus, 2006; Jones and Pfaus, 2014).

In two other KA studies, they intended to lesion the region of interest to study female sexual behavior (Nance, 1983; King and Nance, 1986). They attempted this by KA administration (0.5 and 1 μg of KA) to the lateral septum of Sprague–Dawley rats, which led to a reduction in lordosis quotient (Nance, 1983; King and Nance, 1986). The authors concluded that the dose led to neurotoxicity based on their findings of global changes in other behaviors including hyperventilation and motor activation, despite the 0.5 μg dose of KA not resulting in histological differences distinguishable from those of normal brains (Nance, 1983). This indicated to the author that histological data alone may not suffice to verify the absence of neurotoxicity from the pharmacological manipulation. They did additionally find reduced overall size of the dorsolateral part of the lateral septum, and cell loss in the CA3 and CA4 regions of the hippocampus, possibly because the lateral septum projected to these areas (Nance, 1983). Future studies may want to include additional evidence to verify the absence of neurotoxicity such as from structural imaging, histology of surrounding regions, and electron microscopy to identify ultrastructural changes. Virtually all glutamate in sexual behavior studies are devoid of all of this information, despite the fact that we have convincing data to do so decades ago.

No studies exist, to our knowledge, that have investigated the role of mGluRs in female sexual behavior. Given recent evidence that mGluRs regulate the communication between electrical synapses that lead to neuroplasticity changes during development and in pathology, this is an area of research waiting to be mined (reviewed in Cachope and Pereda, 2020). Furthermore, recent developments have enabled the precise control of glutamate receptors *in vivo* such as through a light-activated KA receptor (Levitz et al., 2016). Researchers transduced V1 neurons of the visual cortex in adult mice with a virus expressing the light-activated KA receptor (Levitz et al., 2016). Upon delivering light to these neurons to activate KA receptors, the researchers were able to increase neuron firing.

### Potential Role of Glutamate in Steroid-Independent Female Sexual Behavior

Sex steroid hormones drive sexual behavior through their cognate receptors (reviewed in Rudzinskas et al., 2019). However, aside from their cognate ligands, other ligands can bind to these receptors. For instance, several of these other ligands developed as drugs (reviewed in Madauss et al., 2007; Farzaneh and Zarghi, 2016; Narayanan et al., 2018). Sex steroid hormones can also act without their cognate receptor, such as the testosterone activation of transient receptor potential melastatin 8, which influenced the frequency of mounting and sexual satiety (Mohandass et al., 2020). Although no studies have shown direct binding of glutamate ligand to steroid receptors, a few studies have suggested indirect interactions. First, in hippocampal neurons, androgen receptor activation changed how glutamate affected the intracellular calcium response (Foradori et al., 2007) and that glutamate had antagonistic effects on androgen receptor (Shannon et al., 2019). Furthermore, glutamate decarboxylase 65 converts glutamate to GABA, and it regulated androgen receptor binding to nuclear proteins in the context of prostate cancer (Gao et al., 2019). In terms of the progesterone receptor, only one study appeared to show possible interactions with glutamate. This study demonstrated that AMPA receptor colocalized with the progesterone receptor in guinea pig hypothalamus (Warembourg and Leroy, 2002).

More cogent evidence exists for estrogen receptor interactions with glutamatergic signaling. In the context of female sexual behavior, mGluR1a directly interacted with estrogen receptor, and this regulated lordosis through a protein kinase C mechanism (reviewed in Dewing et al., 2007, 2008). Through co-immunoprecipitation experiments in hypothalamic astrocytes, Kuo et al. (2009) showed a direct interaction between mGluR1a and estrogen receptor-alpha. One additional study demonstrated an interaction of estrogen receptor with mGluR5 and mGluR3 in female rat striatal neurons (Grove-Strawser et al., 2010). Readers can retrieve extra material on this topic from a review on the relationship between estrogen receptors and mGluR from the perspective of drug addiction (reviewed in Tonn Eisinger et al., 2018). Glutamate ligand interactions with steroid hormone receptors pose new directions for examination in the context of steroid-independent sexual behavior.

In females, sexual experience significantly increases receptivity, neurogenesis in the accessory olfactory bulb, nitric oxide synthase expression in the mPOA, dopamine release in the NAc, and dendritic spine density in the NAc (reviewed in Herrera-Morales et al., 2019; Marco-Manclus et al., 2020). However, no studies exist to our knowledge that has specifically examined how sexual experience would impact female receptivity by glutamate systems. Among the few studies that did disclose the sexual experience status of the females, some of them demonstrated contrasting results to other studies that administered the same compounds. For example, in sexually experienced Long–Evans rats, 200 ng of MK801 administered to the VTA raised their lordosis quotients (Frye et al., 2020), which conflicted with another study administering 0.05–0.4 mg/kg MK801 to Sprague–Dawley rats (Fleischmann et al., 1991). Another example refers to the study that noticed a reduced lordosis quotient upon infusing 3.39 nmol of NMDA to the VMH of Long–Evans rats (Georgescu and Pfaus, 2006), which clashed with the study that observed an increased lordosis quotient with 40 mg/kg NMDA injected intraperitoneally to the same type of rat (Hsu et al., 1993). In many of the studies we have reviewed, the sexual experience status of the female rodents was not disclosed, and it may be worth keeping in mind that differences in sexual experience may contribute a significant factor in identifying the role of glutamate in sexual behavior.

As with males, females display different sexual endophenotypes. In female Wistar rats, there are those that avoid males, those that approach males, and an intermediate group (Snoeren et al., 2011). It would be worth investigating if a potential link exists between glutamate and these differing endophenotypes and its potential role in affecting female sexual behavior independent of gonadal sex steroid hormones.

## Glutamate and Sexual Behavior in Non-rodent Species

In addition to rodents, studying sexual behavior in other animals generates tremendous value. For example, rams offer unique insights into sexual behavior due to our abundant knowledge of their reproduction derived from their long history of domestication (reviewed in Perkins and Roselli, 2007). Some components of ram male sexual behavior match those of rodents, including anogenital sniffing, vocalizations, mounting, intromission, and ejaculation (reviewed in Perkins and Roselli, 2007). Notable differences to rodent male sexual behavior include pawing and nibbling the ewe's flank, as well as flehmen (elevating their head and retraction of the upper lip) (reviewed in Perkins and Roselli, 2007). Only one study investigated the role of glutamate in ram male sexual behavior. Male Dorper rams demonstrated increased male sexual behavior (both appetitive and consummatory) after intramuscular injections of 7 mg/kg glutamate (Calderón-Leyva et al., 2018).

Another animal model, Japanese quails, which humans domesticated since the twelfth century, share similar neuroendocrine systems with mammals and display easily observable sexual behavior during captivity (reviewed in Ball and Balthazart, 2010). The male sexual behavior begins with crowing (mating calls to attract females) and rhythmic contractions of the cloacal gland (similar to non-contact erections in mammals) (reviewed in Ball and Balthazart, 2010). This is followed by the males grabbing the female's neck feathers, mounting, and attaching his cloaca onto the female's cloaca for sperm transfer (Ball and Balthazart, 2010). In these male Japanese quails, glutamate is released in the medial preoptic nucleus during mating (de Bournonville et al., 2017). In respect to targeting GluRs, Seredynski et al. (2015) administered 100 μg of several drugs to the third ventricle of male Japanese quails and determined their influence on male sexual behavior (Seredynski et al., 2015). Both MPEP (mGluR5 antagonist) and LY341495 (mGluR2/3 antagonist) did not affect male sexual behavior. The only changes resulted from administering LY367385 (mGluR1 antagonist), which inhibited rhythmic cloacal sphincter movements.

Above all, the role of glutamate in sexual behavior of non-rodent species remains largely abstruse, despite decades of research in this area on rodents. A recent article shared the same worry as us and postulated this to be the significant cause of the pervasive failure of translatability and reproducibility in behavioral research (reviewed in Mathuru et al., 2020). No one animal model can fully recapitulate the human complexity of sexual behavior. Therefore, compiling evidence from a diverse range of animal models, each representing some part of human sexual behavior, would bring us closer to truly understand it.

As with rodent studies, ascertaining glutamate roles in steroid-independent mechanisms of sexual behavior advances as enthralling future work, particularly considering several non-rodent species exhibit steroid-independent sexual behavior. These encompass the inter-individual variation responses to castration shown in dogs (Beach, 1970), rhesus monkeys (Phoenix et al., 1973), and humans (Zverina et al., 1990). In support of this, 20% of mongrel male cats continued to intromit 15 weeks after castration (Aronson and Rosenblatt, 2008). By the same token, epidemiological studies demonstrate steroid-independent male sexual behavior in humans. For example, sexual desire, erectile function, and sexual function only minimally correlated with testosterone, estradiol, and sex hormone-binding globulin levels in the serum (Cunningham et al., 2015). Another example pertains to the substantial proportion of men remaining sexually active post-castration, with 37% having sex several times per week, and only 8% reported to becoming non-sexual post-castration (Handy et al., 2016). In elderly men with hypoandrogenism that received either testosterone gel or placebo topically applied to the skin, both groups improved sexual desire and erectile function at 12 months (Snyder et al., 2016). Additionally, young male cancer survivors low in sex steroid hormones recuperated their self-reported sexual functioning in both placebo and testosterone groups (Walsh et al., 2019). Whether glutamate relates to sexual behavior after castration, in hypoandrogenism, and cancer-induced hormone changes remains unestablished in humans and other non-rodent species.

In terms of steroid-independent sexual behavior in female non-rodent species, ovariectomy did not abolish copulation in female stump tail macaques from the 7 weeks of testing (Baum et al., 1978; Goldfoot et al., 1978), in female pony mares from the 15 mating tests (Asa et al., 1980), in female New Zealand white rabbits from at least eight mating tests (Beyer et al., 1969), in big brown bats from 59 mating trails (Mendonça et al., 1996), and in common marmoset from at least 32 mating tests (Kendrick and Dixson, 1984). These steroid-independent effects commensurately presented in humans. For example, in two cross-sectional studies, androgen is only limitedly associated with sexual function (Zheng et al., 2020), and bilateral oophorectomy did not affect sexual ideation and function (Erekson et al., 2012). At the same time, progesterone treatment did not improve female sexual dysfunction (Worsley et al., 2016), and female sexual desire does not depend on cyclic hormone changes (Shimoda et al., 2018). As with males, we remain incognizant if glutamate engages significantly in sexual functioning beyond the action of sex steroid hormones.

Ultimately, glutamatergic signaling entangles in male sexual behavior beyond rodents, including in rams and Japanese quails. Burgeoning the studies of non-rodent species in sexual behavior would paint a more comprehensive view on the complexities of human sexual behavior. A better part of glutamatergic signaling in sexual behavior may extend beyond sex steroid hormones, especially given the gargantuan amount of evidence of such steroid independence in the sexual behavior of several species including humans in both males and females.

## Other Mechanisms of Glutamate With Sexual Behavior

Several studies have investigated the role of glutamatergic signaling and sexual behavior in the context of other neurotransmitters. In a *Drosophila* courtship study using RNA interference to knockdown NMDA receptors, nitric oxide was implicated in glutamatergic signaling (Ishimoto and Kamikouchi, 2020). Moreover, dopamine plays a significant role in male sexual behavior, and it is released in the mPOA and the MeA (Dominguez and Hull, 2001). Following that, Dominguez et al. (2004) reverse dialyzed glutamate into the mPOA-increased extracellular dopamine, but when administered with an inhibitor of nitric oxide synthase, this effect disappeared. In further support of this, rodent mating augmented the co-expression of FOS in nitric oxide synthase neurons (Nutsch et al., 2014). Another neurotransmitter, serotonin, is also associated with glutamatergic signaling in sexual behavior. For example, reverse dialyzing selective serotonin reuptake inhibitors into the mPOA attenuated ejaculation-induced glutamate activity (Dominguez and Hull, 2010).

Other neurotransmitters seem to identically engage in the effects of sexual experience. To name a few studies, one study discovered the correlation of varying sexual activity with the number of cells that expressed dopamine receptor 2 (Nutsch et al., 2014). Another evidence came from Roman male rats with high avoidance, which exhibited higher male sexual behavior compared to those with low avoidance (Sanna et al., 2017). A correlation manifested where Roman male rats with high avoidance had higher levels of extracellular dopamine in the mPFC, compared to those with low avoidance (Sanna et al., 2017). This correlation commensurately presented for sexual experience, where sexually experienced rats of both types exhibited higher levels of mPFC dopamine, compared to their sexually naïve counterparts. Finally, dopamine interacted with endocannabinoids to increase sexual motivation in male Wistar rats (Canseco-Alba and Rodríguez-Manzo, 2019). These results posit future work to disentangle how the glutamate system in sexual behavior connects with other neurotransmitters from contexts of brain regions, sexual experience, and strain.

## Future Studies

Through understanding animal sexual behavior, we can develop novel therapeutics for sexual dysfunction. The prevalence of erectile dysfunction advances with aging; researchers estimated that around 20–40% of males between 60 and 69 years of age have experienced erectile dysfunction and that escalates to 50–100% for men in their 70 and 80s (McCabe et al., 2016; Quilter et al., 2017). The Food and Drug Administration has approved pharmaceuticals for male sexual dysfunction; however, these approved phosphodiesterase 5 inhibitors do not treat all males with erectile dysfunction (reviewed in Goldstein et al., 2016). In addition to sexual dysfunction, elucidating the mechanisms of sexual behavior provides a critical step toward understanding the intricacy of sexual diversity. Efforts have begun in this domain, ranging from partner preference tests (reviewed in Balthazart, 2020b), to the search for genes relating to sexual diversity using next-generation sequencing (Ganna et al., 2019), to neuroimaging studies (Poeppl et al., 2016; Wang et al., 2020).

While ample evidence exists for glutamatergic signaling in sexual behavior, we still have much to learn of how glutamate plays a role in both the presence and absence of steroidal hormones. Ventura-Aquino and Paredes (2020) briefly summarized the past 50 years of sexual behavior research in rodents and highlighted brain phenotypes to sexual behavior that require further understanding. This encompassed neurogenesis, neuron size, and dendritic spines, and future studies would benefit from investigating how glutamate may interact with these in the context of sexual behavior.

Another recent review examined behavioral neuroendocrinology as a field and offered recommendations on how future studies can take advantage of new methodologies (reviewed in Balthazart, 2020a). For example, electrophysiology and fiber photometry provide spatiotemporal precise information on neuron activity. One study investigated electrophysiology at the single neuron level in relation to sexual behavior (Matsumoto et al., 2012). They found electrophysiological responses and oscillation changes during male sexual behavior in the fast-spiking interneurons and medium spiny neurons from the NAc. Next, single-cell sequencing can answer questions on cell heterogeneity and stochasticity of gene expression in sexual behavior (reviewed in Kelsey et al., 2017). In tandem with studying neuron behavior, researchers can deploy CRISPR interference, optogenetics, and chemogenetics to directly manipulate glutamate-related neurons. In summary, the future of sexual behavior research holds a plethora of interesting research questions probing the relationship between glutamate and sexual behavior with cutting-edge research tools to address important translational questions of sexual dysfunction and sexual diversity.

## Author Contributions

VC: conceptualization, writing original draft, review, and editing. JP: review, editing, and created the figures. All authors contributed to the article and approved the submitted version.

## Conflict of Interest

The authors declare that the research was conducted in the absence of any commercial or financial relationships that could be construed as a potential conflict of interest.

## Abbreviations

FOS: Fos proto-oncogene
mPOA: Medial preoptic area
VMH: Ventromedial hypothalamus
MMPIP: NAM 6-(4-methoxyphenyl)-5-methyl-3-pyridinyl-4-isoxazolo[4,5-c]pyridin-4(5H)-one
AP7: D-(–)-2-amino-7-phosphonoheptanoic acid
MeA: Medial amygdala
PVN: Paraventricular nucleus
GluR: Glutamate receptor.
